# Establishment and validation of a CT-based clinical deep learning radiomics nomogram for predicting the response to transcatheter arterial chemoembolization in patients with hepatocellular carcinoma

**DOI:** 10.3389/fonc.2026.1773400

**Published:** 2026-04-22

**Authors:** Genxiang Chen, Bo Li, Aijun Liu, Bin Li, Yong Mei, Li Tan, Jing Chen, Zhiyuan Hao

**Affiliations:** 1 Department of Medical Oncology, Shangluo Center Hospital, Shangluo, Shaanxi, China; 2 Department of Liver Surgery, Shangluo Central Hospital, Shangluo, Shaanxi, China; 3 Department of Surgery, Shangluo Traditional Chinese Medicine Hospital, Shangluo, Shaanxi, China

**Keywords:** deep learning, hepatocellular carcinoma, machine learning, radiomics nomogram, transcatheter arterial chemoembolization

## Abstract

**Background:**

Hepatocellular carcinoma (HCC) is a common malignant tumor of the liver worldwide, significantly impacting human health. Transcatheter arterial chemoembolization (TACE) is one of the key treatment modalities for HCC, making accurate prediction of the TACE response (TR) crucial for personalized treatment. This study aimed to develop and validate a clinical deep learning (DL) -based radiomics (Rad) nomogram (CDLRN) using pre-treatment contrast-enhanced computed tomography (CT) images and clinical data to predict TR in patients with HCC.

**Methods:**

Clinical and CT data were retrospectively collected from HCC patients who underwent TACE at the Department of Hepatobiliary Surgery of Shangluo Central Hospital from January 2019 to December 2023. A total of 144 patients met the study criteria and were randomly divided into training and validation cohorts at a ratio of 7:3, resulting in 102 and 42 patients, respectively. TR was assessed in according with the Modified Response Evaluation Criteria in Solid Tumors (mRECIST). Univariate and multivariate logistic regression (LR) analyses were conducted to identify independent risk factors associated with TR. Rad and DL features were extracted from preprocessed arterial-phase CT images. These features and image signatures were constructed through four steps: repeatability evaluation, univariate analysis, least absolute shrinkage and selection operator (LASSO) method, and multivariate LR analysis. A novel CDLRN model was then established to predict TR.

**Results:**

Among all models, the CDLRN significantly outperformed the others (P<0.05). Specifically, the area under the receiver operating characteristic curve (AUC) was 0.958 (95% confidence interval [CI]: 0.916-1.000) for the training cohort and 0.916 (95% CI: 0.835-0.997) for the validation cohort, both significantly higher than individual indicators. The CDLRN model demonstrated a specificity of 0.957, sensitivity of 0.839, and overall accuracy of 89.2% in the training cohort. In the validation cohort, the specificity was 1.0, sensitivity was 0.741, and accuracy was 83.3%. “The calibration curves demonstrated strong concordance between predicted and actual probabilities, underscoring the model’s reliability. Furthermore, decision curve analysis highlighted the substantial clinical utility of the CDLRN model.

**Conclusion:**

The CDLRN showed high discrimination and accuracy in predicting the response to the first TACE in patients with HCC, demonstrating its potential to provide invaluable information for clinical decision-making.

## Introduction

Primary liver cancer is a common cancer worldwide, severely affecting human health (1), it ranks as the sixth most common cancer and the third leading cause of cancer deaths (2, 3). The occurrence of HCC is a series of complex biological processes involving genomics (4), and most HCC patients in China are also highly associated with hepatitis B virus infection (1, 5). The 5-year survival rate for HCC patients has improved, but many are diagnosed at intermediate or advanced stages due to the lack of early symptoms, limiting surgery and worsening prognosis. Treatment strategies include drug therapy, transarterial chemoembolization (TACE), hepatic arterial infusion chemotherapy, local ablation, and liver transplantation (6). Among these, TACE (7, 8) is a key treatment for patients with advanced HCC, especially those classified as Stage B according to the Barcelona Clinic Liver Cancer (BCLC) staging criteria (7). However, tumor heterogeneity leads to variable responses to TACE, resulting in high local recurrence rates and significant prognostic disparities (8, 9). Predicting treatment response before TACE is vital for personalizing HCC treatment and improving survival. When TACE is unlikely to help, choosing alternative therapies promptly is crucial. Therefore, a reliable method to predict TR before TACE is urgently needed for managing intermediate to advanced HCC (10, 11).

Current studies (12, 13) have shown that factors such as tumor size, margin, internal arteries, Child-Pugh grade, pathological stage, microvascular invasion (MVI), and various blood test indices, including alpha fetoprotein (AFP), are closely related to the prognosis of TACE and hold predictive value for patients with unresectable HCC Additionally, some research (14, 15) has confirmed that the methylation of cytokine signaling 1 and 3 is highly correlated with TR. However, the accuracy and sensitivity of traditional prognostic markers for predicting TR in unresectable HCC are often limited and usually require invasive procedures. Moreover, regional differences and comorbidities can also cause discrepancies in clinical predictions, failing to fully capture tumor heterogeneity. This variability can hinder clinical decision-making, worsen prognosis, and make these methods hard to apply widely in practice (13, 16).

In recent years, the rapid advancement of medical statistics and computer software (17, 18) has led to the widespread recognition of medical imaging omics among researchers. This approach uses medical image data, combining data mining and statistical tools to extract high-throughput features for disease prognosis and prediction (18).

Multi-omics prediction models that integrate various omics data and traditional logistic regression (LR) analysis significantly outperform conventional empirical models in accuracy (18–20). Liver cancer research has seen a significant rise in artificial intelligence applications, especially in radiomics (Rad), machine learning, and deep learning (DL). These technologies have been used for differential tumor diagnosis, pathological grading (21), MVI assessment (22, 23), early recurrence (21) prediction of treatment efficacy (24). Studies (20, 22, 24) have shown that Rad offers high accuracy and predictive value for assessing pathological grades, drug responses, asymptomatic survival times, and early recurrence in liver cancer. Additionally, studies (24–26) have demonstrated that DL is valuable in predicting MVI, lymphatic metastasis, postoperative recurrence, and overall prognosis. Therefore, we hypothesize that a model integrating clinical (Clinic), Rad, and DL features preoperatively could improve TR prediction in unresectable HCC patients, evaluate TACE effects, and support individualized treatment strategies to enhance survival. Contrast-enhanced computed tomography (CT) is a common diagnostic tool for primary liver cancer. This study aims to develop an optimal CT-based CDLRN model for preoperative TR prediction. We aimed to validate the predictive performance of this model using the concordance index (C-index), calibration curves, decision curve analysis (DCA), and internal validation sets. A nomogram was used to visualize the CDLRN linear model, improving the readability of prediction results and discussing the model’s clinical feasibility.

## Materials and methods

### Patients

This study was in accordance with the Declaration of Helsinki (revised in 2013), approved by the Ethics Committee of Shangluo Central Hospital (LW2024040), with a waiver for informed consent. The data of 144 inpatients diagnosed with HCC who were unresectable or unwilling to receive curative treatment and initially treated with TACE at Shangluo Central Hospital between February 2019 and December 2023. The enrollment process is illustrated in Appendix E1 and **Supplementary Figure 1**.

### Demographic and clinical characteristics of patients

Demographic and clinical characteristics were obtained from the Hospital Information System (HIS) and included age, sex, leukocyte count, platelet count, prothrombin time, activated partial thromboplastin time, fibrinogen, total bilirubin, alanine aminotransferase, aspartate aminotransferase, creatinine, ascites presence, Child-Pugh score, AFP levels, hepatitis status, cirrhosis status, BCLC staging, and performance status score. Three radiologists (ZY, ZJQ, and LHY), with 8, 9, and 21 years of experience, respectively, evaluated routine semantic features of the CT scans, including maximum tumor diameter, number of tumors, tumor capsule (incomplete/complete), and margin (clear/blurred). Follow-up evaluations occurred one month after the first TACE treatment and every three months thereafter, during which contrast-enhanced CT scans were performed to assess the treatment response.

### TACE treatment and response assessment

TACE treatment and response assessment are described in detail in the Appendix E2. The TR group included patients with a complete response (CR) or partial response (PR), while those with disease progression (PD) or stable disease (SD) were classified as nTR.

### CT examination and image preprocessing

Contrast-enhanced CT is the primary examination method for diagnosing and treating patients with HCC and is the most widely used imaging modality in Rad research. Details of image acquisition and processing are documented in Appendix E3 and **Supplementary Table 1**.

### Feature extraction and selection

**Figure 1:**
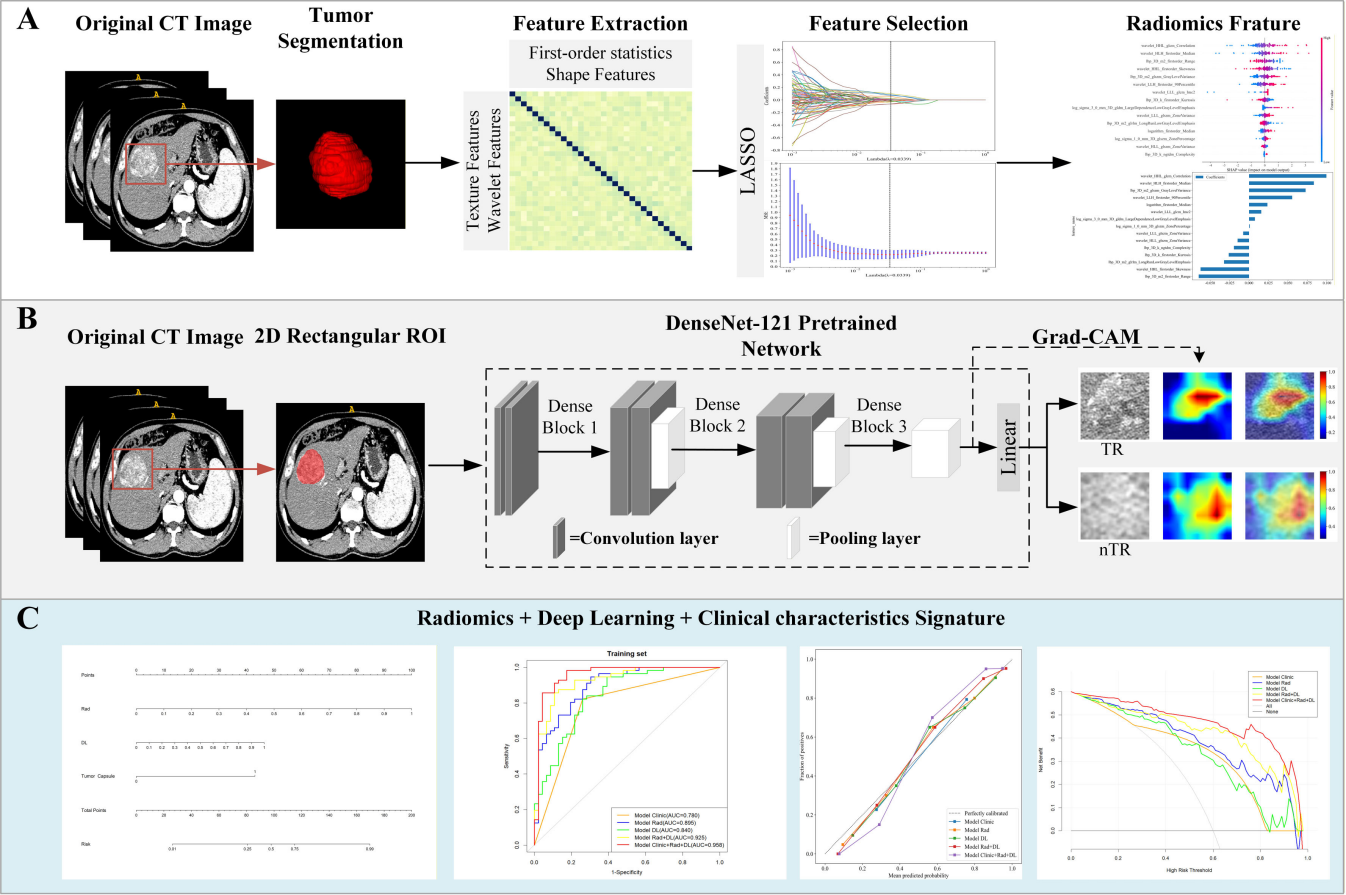
Overview of the study pipeline. **(A)** Baseline construction process of the traditional machine learning LR model. **(B)** Construction process of the deep learning model, which uses two-dimensional arterial phase computed tomography images as input for the pre-trained model based on the classic convolutional neural network (DenseNet-121). The predicted probabilities for TR and nTR are the outputs. Additionally, Grad-CAM is utilized to visualize the model’s decision-making process. **(C)** Development of the CDLRN for predicting TR in patients with unresectable hepatocellular carcinoma undergoing TACE treatment. CT, computed tomography; LR, logistic regression; TR, TACE response; nTR, non-TACE response; Grad-CAM, gradient-weighted class activation mapping; CDLRN, clinical deep learning radiomics nomogram; TACE, transarterial chemoembolization.

**Figure 1A** shows the workflow of this study. To enhance accuracy and stability, the TR after the first TACE treatment was independently evaluated by two experienced radiologists (A and B) according to the mRECIST. A total of 1,834 quantitative radiomic features were extracted from the VOIs using the Pyradiomics package (version 3.0.1). The deep learning features were extracted using the pre-trained DenseNet-121. Detailed information on the Rad and DL features extraction process can be found in Appendix (E4, E5) and **Supplementary Table 2**.

### DL and Rad signature building

We selected the maximum 2D ROI plane of the tumor for analysis. Logistic regression analysis was performed on selected Rad features and DL features. Define the individual probability value of the output as signature, so as to obtain the DL signature and Rad signature. Detailed information on the Rad and DL signature building can be found in Appendix (E4, E5).

Using gradient-weighted class activation mapping (Grad-CAM), we visualized the DL model by weighting the gradient information from the last convolutional layer of the CNN. This produced a class activation map highlighting significant areas, improving the model’s interpretability. **Figure 1B** shows the entire DL model development process.

### CDLRN construction

For the screening of clinical features, continuous variables that conform to a normal distribution are described using the mean and standard deviation, with intergroup comparisons performed using the Student’s t-test. For continuous variables with a skewed distribution, they are presented as the median and interquartile range, and intergroup comparisons are conducted using the Mann–Whitney U test. Categorical variables are expressed as proportions and compared using either the Chi-square test or Fisher’s exact test.

We combine the screened clinical features, Rad signatures, and DL signatures to develop TR prediction models using four different machine learning methods: LR, support vector machine (SVM), multilayer perceptron (MLP), and random forest (RF). The procedure for selecting the LR model is described in detail in the Appendix E6. The random seed was set to 100 in the training group. We assessed model performance using average accuracy, recall, precision, F1 score, positive predictive value, and area under the curve (AUC) across various machine learning algorithms. The best method was chosen to construct a TR model, which was then validated in a separate validation group. Finally, the best LR model was chosen to build the CDLRN model, as shown in **Figure 1C**, along with a nomogram for model visualization. The efficiency of this model was significantly enhanced compared to other models, as confirmed by DeLong testing (p < 0.05); the specific results are detailed in **Supplementary Figure 2**.

### Statistical analysis

The Shapiro-Wilk test assessed normality for continuous variables. Normally distributed variables are presented as means with standard deviations, and differences between groups were compared using Student’s t-test. For skewed variables, medians and interquartile ranges are reported, with the Mann-Whitney U test for cohort comparisons. Categorical variables are expressed as rates and composition ratios, with differences analyzed using the Pearson chi-squared test or Fisher’s exact test. A significance level of p<0.05 was set. Independent predictors of TR were identified through univariate and multivariate analyses, including the LASSO regression model. Logistic regression analysis used a stepwise backward approach based on the Akaike Information Criterion (AIC) to build a diagnostic prediction model. The Rad-signature and DL-signature were included in the clinical model to form a combined model. Predictive performance was evaluated using accuracy, sensitivity, specificity, positive predictive value (PPV), negative predictive value (NPV), and AUC. R software was used for model fitting and nomogram generation, with accuracy verified using calibration curves and the Hosmer-Lemeshow test. The DeLong test (27) compared AUC values, and clinical utility was assessed through DCA, validated in a separate group. Two-sided p<0.05 was considered statistically significant. All statistical analyses were performed using R software (version 4.1.2; http://www.r-project.org/).

## Results

### Demographic and clinical characteristics of patients

A total of 144 patients were enrolled in this study, including 83 with TR and 61 with nTR. The average age was 59.8 ± 12 years, with a male-to-female ratio of 3.65:1 (113 males and 31 females). Among the participants, 121 had hepatitis B cirrhosis, and 92 had multiple tumors. Patients were randomly divided into a training group (102) and a validation group (42), with no significant differences between the two cohorts (P > 0.05). Baseline characteristics are summarized in **Table 1**. Univariate regression analysis was performed in the training group, resulting in five selected variables (Ascites, ECOG, Child-Pugh score, margin, and tumor capsule) for multivariate LR analysis, the findings are summarized in **Table 2**. Notably, the tumor capsule variable showed a significant difference in TR and nTR between the two patient groups (P < 0.05) and was identified as an independent predictor of TR, which was used to construct a clinical model.

**Table 1:** Clinical characteristics of patients in the training and validation cohorts.

Variables	Training cohort (n=102)	Validation cohort (n=42)	P value
Age (years), mean (SD)	59.04 (11.94)	61.57 (13.06)	0.256
Gender, n (%)			0.985
Male	80 (78.43)	33 (78.57)	
Female	22 (21.57)	9 (21.43)	
WBC*10^9^, median (IQR)	5.21 (3.26, 6.89)	4.93 (3.39, 7.10)	0.679
PLT*10^9^, median (IQR)	152.0 (90.0, 217.2)	127.5 (84.0, 156.8)	0.091
PT, mean (SD)	13.91 (2.60)	18.29 (23.32)	0.686
APTT, mean (SD)	81.31 (20.16)	79.50 (15.31)	0.763
Fib, median (IQR)	2.85 (2.15, 3.56)	2.66 (1.95, 3.47)	0.496
TBIL, median (IQR)	20.15 (13.25, 31.85)	21.85 (16.88, 35.30)	0.207
ALT, median (IQR)	38.5 (20.0, 64.3)	32.5 (25.5, 62.8)	0.886
AST, median (IQR)	61.5 (30.0, 100.3)	53.0 (33.8, 97.5)	0.738
Cr, median (IQR)	57.0 (49.0, 72.0)	59.5 (50.0, 67.3)	0.974
AFP, median (IQR)	436.55 (85.90, 1918.28)	290.05 (45.68, 1891.78)	0.667
TD, mean (SD)	7.98 (3.83)	7.38 (3.57)	0.389
TN, n (%)			0.065
Single	32 (31.4)	20 (47.6)	
Multiple	70 (68.6)	22 (52.4)	
Ascites, n (%)			0.891
Absent	74 (72.5)	30 (71.4)	
Present	28 (27.5)	12 (28.6)	
HBsAg, n (%)			0.269
Positive	83 (81.37)	38 (90.48)	
Negative	19 (18.63)	4 (9.52)	
Cirrhosis, n (%)			0.243
Absent	49 (48.04)	15 (35.71)	
Present	53 (51.96)	27 (64.29)	
BCLC stage, n (%)			0.397
A	28 (27.45)	16 (38.10)	
B	68 (66.67)	23 (54.76)	
C	6 (5.88)	3 (7.14)	
Child-Pugh, n (%)			0.569
A	40 (39.22)	19 (45.24)	
B	36 (35.29)	11 (26.19)	
C	26 (25.49)	12 (28.57)	
ECOG, n (%)			0.688
0	57 (55.88)	25 (59.52)	
1	45 (44.12)	16 (40.48)	
Tumor Capsule, n (%)			0.709
Incomplete	44 (43.14)	16 (38.10)	
Complete	58 (56.86)	26 (61.90)	
Margin, n (%)			0.101
Blur	59 (57.84)	18 (42.86)	
Clear	43 (42.16)	24 (57.14)	

WBC, white blood cells; PLT, platelet; PT, prothrombin time; APTT, Activated partial thromboplastin time; Fib, fibrinogen; TBIL, total bilirubin; ALT, alanine aminotransferase; AST, Aspartate Aminotransferase; AFP, alpha fetoprotein; Cr, serum creatinine; TD, tumor diameter; TN, tumor number; ECOG, Eastern Cooperative Oncology Group.

**Table 2:** Univariate and multivariate logistic regression analyses of clinical characteristics for TR in the training cohort.

Variables	Univariate analysis		Multivariate analysis	
	P value	OR (95%CI)	P value	OR (95%CI)
Age, (years)	0.198	1.004 (0.999-1.010)		
Gender	0.282	1.183 (0.916-1.528)		
WBC*10^9^	0.881	0.996 (0.948-1.045)		
PLT*10^9^	0.957	0.997 (0.993-1.002)		
PT, (s)	0.440	1.011 (0.988-1.035)		
APTT, (s)	0.258	1.003 (0.999-1.007)		
Fib, (g/L)	0.640	1.029 (0.931-1.138)		
TBIL, (μmol/L)	0.226	0.988 (0.970-1.007)		
ALT, (U/L)	0.538	1.001 (0.998-1.005)		
AST, (U/L)	0.244	0.998 (0.995-1.001)		
Cr, (μmol/L)	0.242	1.003 (0.999-1.008)		
AFP, (ng/mL)	0.417	1.000 (1.000-1.000)		
TD, (cm)	0.366	0.980 (0.945-1.017)		
TN	0.083	0.904 (0.822-0.995)		
Ascites	**0.005**	0.273 (0.128-0.582)	0.148	0.326 (0.091-1.166)
HBsAg	0.155	1.371 (0.951-1.976)		
Cirrhosis	0.680	0.893 (0.568-1.404)		
BCLC stage	0.405	0.840 (0.595-1.186)		
Child-Pugh	**0.014**	0.637 (0.471-0.860)	0.945	0.967 (0.469-1.994)
ECOG	<**0.001**	0.324 (0.183-0.572)	0.136	0.355 (0.113-1.113)
Tumor Capsule	<**0.001**	3.833 (2.250-6.534)	**0.003**	5.823 (2.208-15.348)
Margin	<**0.001**	5.286 (2.683-10.412)	0.176	2.522 (0.819-7.768)

OR, odds ratio; CI, confidence interval; The bold values mean the P value<0.05.

### Rad and DL signatures validation

**Figure 2:**
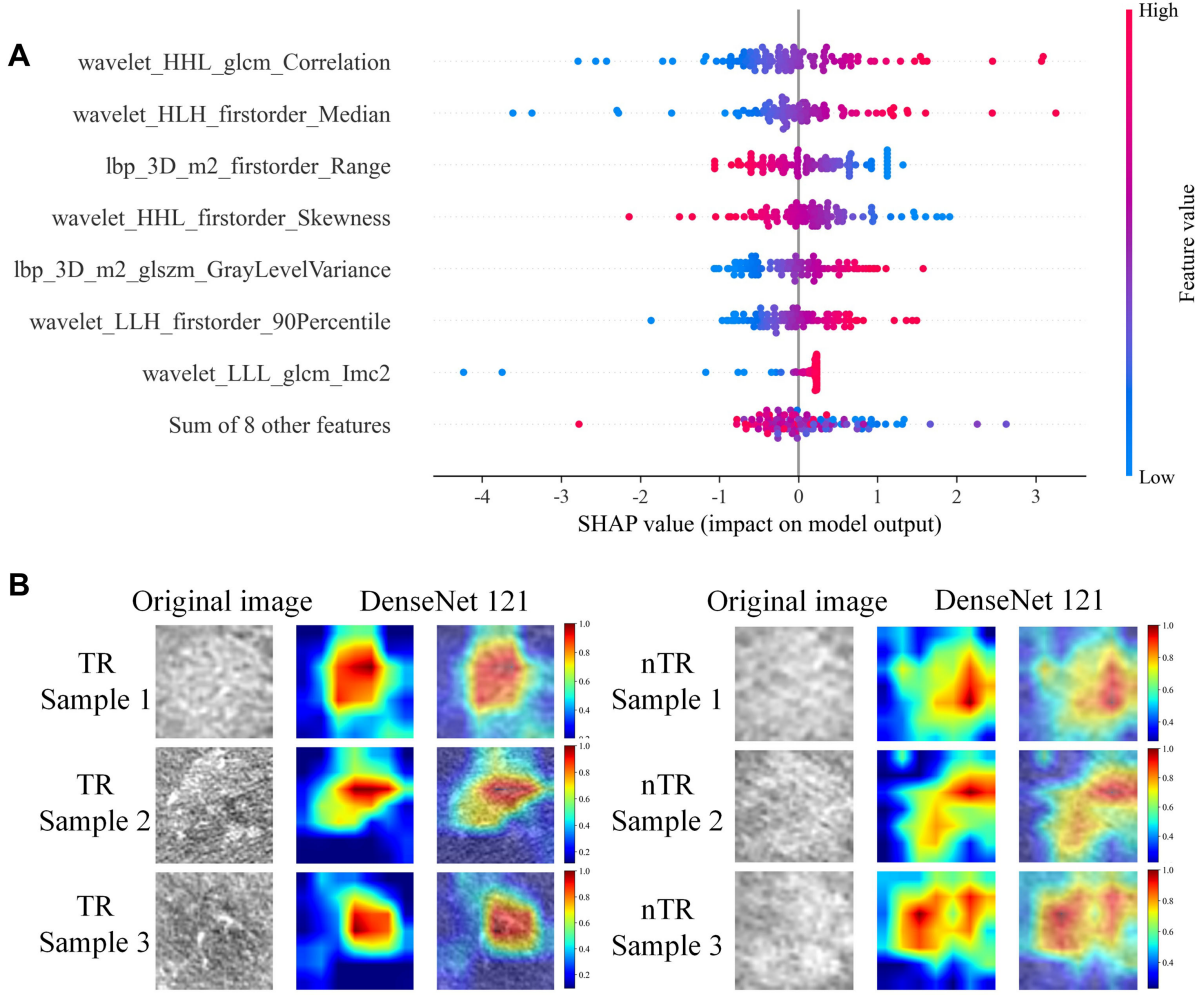
**(A)** SHAP summary plot of the radiomics signature model, illustrating feature relevance and the contribution of combined features to the model’s predictive performance. **(B)** Areas of interest identified by the deep learning models in CT image analysis of hepatocellular carcinoma. SHAP, Shapley Additive exPlanation.

After a thorough feature screening process, the features related to TR in the training group were reduced to 15 Rad features and 4 DL features. These features constructed the Rad signature and DL signature, respectively. The detailed process for constructing these signatures and the selected features are provided in Appendix E4 and **Supplementary Table 2**. For the construction of the Rad signature model, we used Shapley Additive exPlanation (SHAP) values (28) to visually explain feature importance for the LR machine learning model (**Figure 2A**). SHAP summarized the data for each patient, with each point on the graph representing an individual patient, colored by feature value from low (blue) to high (red), and ranked by importance. **Figure 2A** shows that the wavelet_HHL_glcm_Correlation feature is the most significant for distinguishing TR from nTR, with an increasing trend in model output corresponding to higher feature values. To enhance the interpretability of the DL model’s output, we applied Grad-CAM for visualization (**Figure 2B**) (29). This technique revealed obvious differences in key areas identified by each model within the sample. Notably, the Rad signature exhibited superior predictive performance compared to the DL signature. The C-index for TR prediction was 0.895 (95% confidence interval [CI], 0.834-0.957) for the Rad signature and 0.840 (95% CI, 0.763-0.917) for the DL signature. Although the Rad signature outperformed the DL signature, the combined deep learning radiomics (DLR) signature, which incorporated both Rad and DL features, achieved the best performance with a C-index of 0.925 (95% CI, 0.873-0.977). In the validation group, all models demonstrated strong predictive ability (**Table 3**).

**Table 3:** The performance of different models in the training and validation cohorts.

Model		AUC (95% CI)	Accuracy	Sensitivity	Specificity	PPV	NPV
Clinical model	Training	0.780 (0.699-0.862)	0.784	0.821	0.739	0.793	0.773
	Validation	0.826 (0.701-0.951)	0.833	0.852	0.80	0.885	0.750
Rad signature	Training	0.895 (0.834-0.957)	0.833	0.946	0.696	0.791	0.914
	Validation	0.731 (0.573-0.889)	0.714	0.778	0.60	0.769	0.562
DL signature	Training	0.840 (0.763-0.917)	0.784	0.821	0.739	0.793	0.773
	Validation	0.733 (0.562-0.905)	0.714	0.667	0.80	0.857	0.571
DLR signature	Training	0.925 (0.873-0.977)	0.873	0.857	0.891	0.906	0.737
	Validation	0.726 (0.564-0.888)	0.738	0.778	0.667	0.808	0.625
CDLRN	Training	0.958 (0.916-0.999)	0.902	0.857	0.957	0.960	0.846
	Validation	0.916 (0.835-0.997)	0.857	0.778	0.998	0.989	0.714

AUC, area under the receiver-operating-characteristic curve; CI, confidence interval; PPV, positive predictive value; NPV, negative predictive value;.

Rad, radiomics; DL, deep learning; DLR, deep learning and radiomics; CDLRN, clinical and deep learning and radiomics nomogram.

### Performance and validation of CDLRN

**Figure 3:**
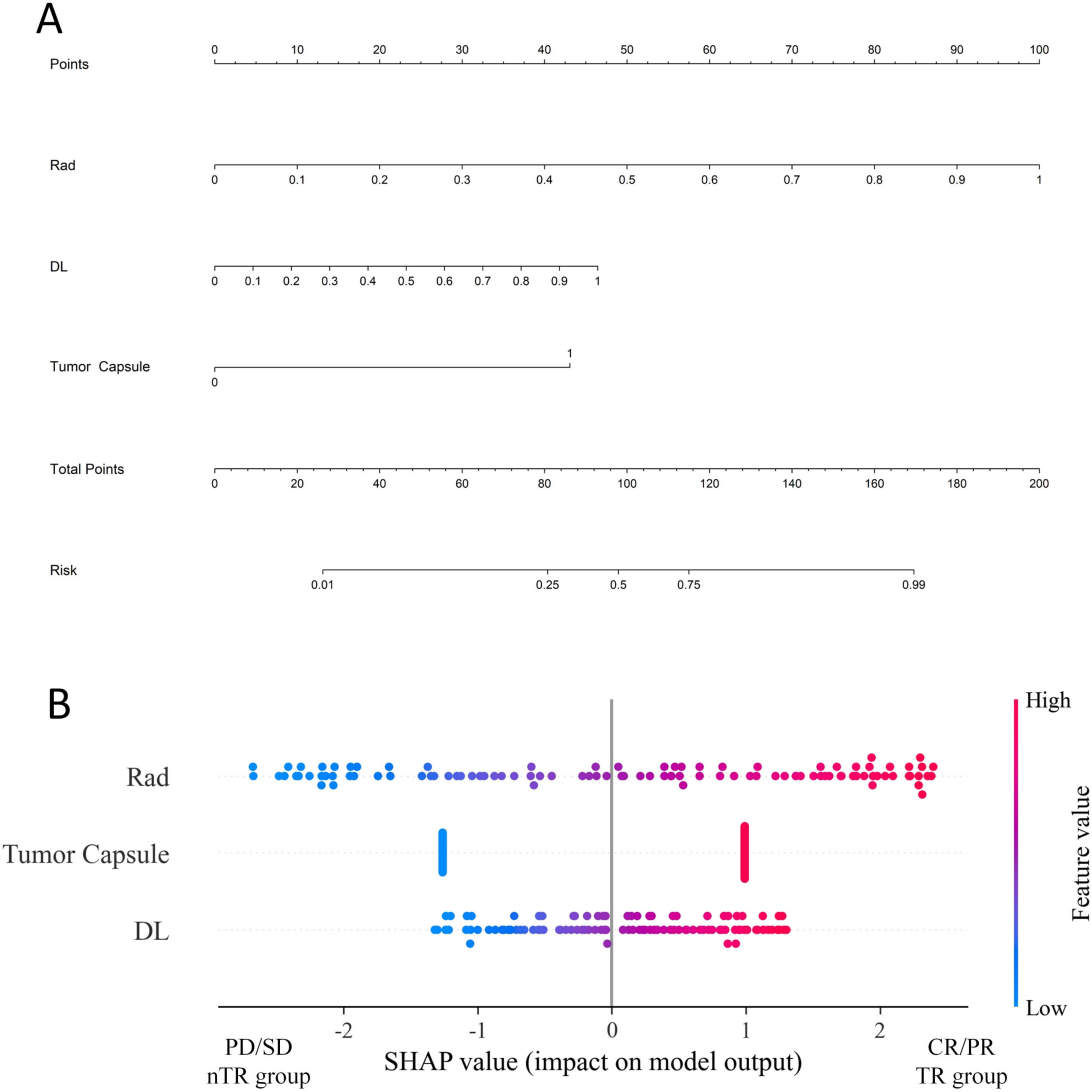
**(A)** Predictive nomogram for estimating the probability of TR. **(B)** SHAP summary plot of the radiomic-clinical model, demonstrating feature relevance and the contribution of combined features to the model’s predictive performance. TR, TACE response; SHAP, Shapley Additive exPlanation.

**Figure 4:**
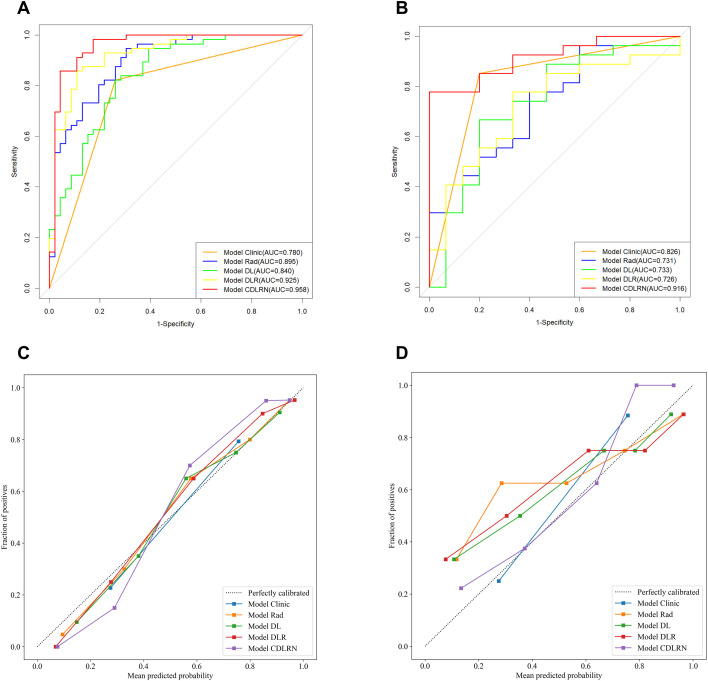
Receiver operating characteristic curves for the five models in the training cohort **(A)** and validation cohort **(B)**. Calibration curves for the five models in the training cohort **(C)** and validation cohort **(D)**.

**Figure 5:**
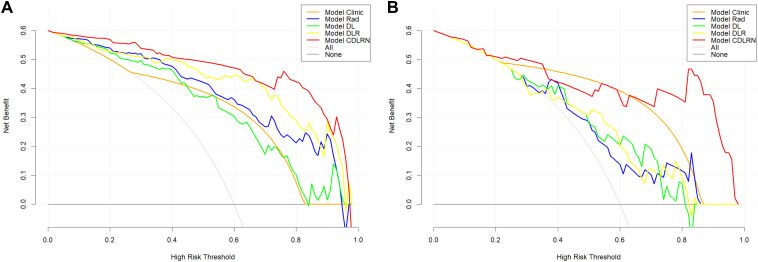
Decision curve analysis for the CDLRN, deep learning signature, radiomics signature, deep learning radiomics signature, and clinical model in the training cohort **(A)** and validation cohort **(B)**. CDLRN, clinical deep learning radiomics nomogram.

In the training group, the Rad-based signature, DL-based signature, and Tumor Capsule were identified as independent risk factors for TR. We constructed a combined model, termed the CDLRN, using backward stepwise LR based on the minimum AIC for these factors (**Figure 3A**). SHAP provided a nomogram illustrating the range and distribution of feature importance in relation to model output. Notably, the Rad signature emerged as the most important feature for distinguishing between TR and nTR classifications (**Figure 3B**). The AUC values for the Clinical Model, Rad signature, DL signature, DLR model, and CDLRN in the training group were 0.780 (95% CI, 0.699-0.862), 0.895 (95% CI, 0.834-0.957), 0.840 (95% CI, 0.763-0.917), 0.925 (95% CI, 0.873-0.977), and 0.958 (95% CI, 0.916-0.999), respectively. Similarly, each model maintained a high AUC in the validation group (**Table 3**). This demonstrated that the CDLRN model had superior predictive performance among all models (**Figure 4A**). By integrating Rad and DL features into the clinical model, we enhanced its predictive accuracy (**Table 3**). Furthermore, in the validation group, the performance of the CDLRN model was significantly better than that of the other models (**Figure 4B**). Both in the training set and the validation group, the calibration curves for all models showed good consistency between the TR prediction of CDLRN and the actual observations (p > 0.05) (**Figures 4C, D**). Additionally, DCA revealed that across all cohorts, the CDLRN provided a greater net benefit than the other models at relevant thresholds (**Figures 5A, B**).

## Discussion

TACE is a treatment for patients with unresectable intermediate and advanced HCC, serving as a conventional therapeutic approach (30–32). Its primary mechanism involves embolizing the tumor’s blood supply, which induces ischemia and subsequent necrosis, thereby inhibiting tumor progression (32–34). However, tumor heterogeneity, spatial structure variations, and individual differences can lead to significant differences in tumor behavior. As a result, some HCC patients may be refractory to TACE (35, 36), leading to local tumor recurrence or metastasis. Therefore, accurately assessing the objective response of the tumor prior to the first TACE treatment is crucial for timely adjustments in therapeutic decisions. Furthermore, the early and accurate prediction of treatment response and prognosis has certain reference value for selecting appropriate treatment plans for individual patients. As medical science advances, understanding tumor heterogeneity has improved, but information on intratumoral heterogeneity from routine exams remains limited. In this study, we proposed and validated a non-invasive CDLRN model based on preprocessed arterial abdominal CT images, using DL and Rad to analyze image features. This model aims to accurately classify HCC patients into nTR and TR groups preoperatively. Our findings suggest the CDLRN model can help clinicians identify the objective response of patients to TACE treatment at an early stage and has certain clinical significance in the treatment selection of HCC patients.

HCC is clinically heterogeneous, requiring accurate TR and prognosis predictions to select appropriate therapies. AFP, a biomarker strongly correlated with HCC, is widely used for early diagnosis and monitoring (37–39). It serves as an important indicator of tumor activity and is often considered an independent risk factor for early recurrence after HCC resection. Additionally, some studies (40, 41) have (42, 43) reported a negative correlation between serum AFP levels and overall survival (OS) time following TACE. However, AFP has limitations in prognostic prediction for HCC, as its measurement can be invasive and may not accurately predict outcomes for small tumors (≤3 cm). In our study, AFP was not a reliable predictor of objective response to TACE; instead, the Tumor Capsule feature emerged as a significant predictor. This discrepancy may stem from patient differences and varying sample sizes in previous studies. Our findings show that Tumor Capsule is highly correlated with TR (P < 0.05) and serves as a non-invasive, easily obtainable predictor of TACE outcomes.

This conclusion is supported by other reports (32, 44, 45). Overall, Tumor Capsule was found to be an independent predictor of TR, achieving an AUC of 0.780 (95% CI: 0.699-0.862). This study highlighted a novel machine learning approach for Rad analysis, which quantitatively extracted high-throughput image features from medical images for computer-aided data analysis, allowing prediction of treatment responses for various tumors. Prior research indicates that Rad analysis has potential predictive value in liver cancer (46, 47), lung cancer (48), cholangiocarcinoma (49), gastric cancer (50), and colorectal cancer (51). Studies (22, 25, 47) have shown that although clinically relevant risk factors provide predictive insights for HCC patients prior to TACE, they often overlook critical imaging information. In contrast, Rad analysis offers detailed insights into intratumoral heterogeneity that may not be discernible to radiologists. Certain texture features, such as those derived from the gray level co-occurrence matrix (GLCM) and gray size zone matrix (GLSZM), can effectively identify TR.

In our study, we developed a Rad model with an AUC of 0.895 (95% CI: 0.834-0.957). Notably, the 15 Rad features used in our analysis included conversion factors, particularly Laplacian of Gaussian (LoG) and wavelet-based features, which provided deeper insights into tumor heterogeneity. In addition, in some reports (52–54), SHAP analysis has aided in elucidating the prediction logic of the model by assessing the contribution of each feature to the prediction outcomes. We ranked the Rad features based on their importance and selected the top eight for SHAP analysis visualization, helping to clarify the influence of each feature on the Rad signature model’s outputs (**Figure 2A**).

In this study, we used transfer learning with the DenseNet-121 CNN architecture for DL feature extraction. Unlike traditional manual measurement features, DenseNet-121 is widely used in vision applications due to its excellent performance and efficient parameter usage. This structure allows for the extraction of task-specific depth information from the neural network’s hidden layers, even though the DL features themselves lack specific physical meanings. The features captured by this DL algorithm can effectively predict early recurrence, metastasis after liver cancer resection, and survival outcomes for resectable HCC (38, 55). Previous studies (21, 56) have demonstrated that DL offers valuable insights into the tumor microenvironment, especially regarding spatial heterogeneity and TACE sensitivity. In our study, DL features predicted TR well, with AUC values of 0.84 in the training group and 0.73 in the validation group. There was no significant difference in performance between the DL prediction model, the clinical model, and the Rad signature (P > 0.05). However, the performance of the combined CDLRN model—integrating the DL signature with the Rad signature and clinical features—outperformed any individual model. Notably, Grad-CAM visualization has been shown in previous studies to intuitively display complex tumor structures. Since both tumor boundaries and internal structure are critical areas of interest in HCC research, we employed Grad-CAM to visualize these aspects in our study, aiding in the differentiation between TR and nTR. The visualizations revealed that nTR tumors exhibited irregular shapes, unclear boundaries, and complex intratumoral heterogeneity compared to TR tumors, further validating the effectiveness of the DL signature.

We found that the predictive capabilities of the CDLRN model in both the training and validation groups were superior to any other model (p < 0.05), with AUCs of 0.958 and 0.916, respectively. Previous studies (57) have identified multiple clinical or molecular risk factors associated with TR; however, the results from these indicators (58, 59) can be inconsistent. This variability may be attributed to differing distributions of clinical characteristics among patients. The CDLRN model addresses this issue by incorporating high-dimensional imaging features that specifically quantify intratumoral heterogeneity, thus enhancing predictive performance. Constructed from 15 radiomic features, 4 DL features, and 1 clinical factor, these selected imaging features were tested for correlation and were found to be weakly or uncorrelated. Furthermore, the clinical utility of constructing a CDLRN model through decision curve evaluation can provide reference value for the selection of treatment options for HCC patients. Therefore, the CDLRN model has some reference value for whether to undergo TACE treatment in HCC patients.

This study had several limitations. Firstly, it was a single-center retrospective study with a small sample size, introducing bias. The CDLRN model’s generalizability should be validated in larger, multi-center prospective studies. Secondly, DL feature extraction used two-dimensional (2D) imaging from the largest tumor cross-section instead of 3D imaging, potentially missing critical information from the tumor’s upper and lower layers. Future research should focus on comprehensive 3D analysis. Thirdly, we did not conduct a survival analysis, and longer patient follow-up is needed for meaningful survival data. Continuous monitoring of outcomes is essential for assessing TACE prognosis. Lastly, the CDLRN model lacked biological features, leaving the significance of DL features unclear. Future studies integrating Rad with pathomics or genomics may provide deeper insights into HCC’s microscopic characteristics.

## Conclusion

In summary, we developed and validated a novel, non-invasive CT-based model using DL and Rad analysis to predict treatment response in HCC patients before TACE. The CDLRN model effectively combines imaging features and clinical factors, providing a new idea for HCC treatment. However, further research is still needed to confirm its clinical value.

## Data Availability

The raw data supporting the conclusions of this article will be made available by the authors, without undue reservation.
