# Supplementary material for: Establishment and validation of a CT-based clinical deep learning radiomics nomogram for predicting the response to transcatheter arterial chemoembolization in patients with hepatocellular carcinoma
Source: Front Oncol. 2026 Apr 22;16:1773400. doi: 10.3389/fonc.2026.1773400 (PMC13147201; doi:10.3389/fonc.2026.1773400)
Supplement: Supplementary file 1 [file DataSheet1.docx]

**Establishment and validation of a CT-based clinical deep learning radiomics nomogram for predicting the response to transcatheter arterial chemoembolization** **in patients with hepatocellular carcinoma**

**Electronic Supplementary Material**

**1. Supplementary Methods**

**Appendix E1 Patient Recruitment**

This retrospective study included 144 consecutive patients with unresectable hepatocellular carcinoma (HCC) who received TACE treatment according to the following inclusion and exclusion criteria.

Inclusion criteria were as follows: (1) Confirmed diagnosis of HCC with no previous HCC-related therapy and unresectable or unwilling to undergo curative treatment. (2) Complete and clear enhanced CT images within one week prior to TACE treatment; (3) Initially treated with TACE monotherapy; and (4) The follow-up for more than six months. Exclusion criteria included: (1) Have contraindications to TACE; (2) Receiving other treatments other than TACE within 6 months after the initial TACE treatment, such as radiotherapy, chemotherapy, targeted therapy, immunotherapy, or ablation; (3) Intrahepatic vascular invasion; and (4) Extrahepatic or lymph node metastasis. After applying these detailed inclusion and exclusion criteria, 144 patients were finally included in the study. The study included unresectable or unwilling to undergo radical treatment and initially HCC patients treated with TACE. In other words, all the stages except for the terminal stage (CNLC stage IV and BCLC stage D) were screened for inclusion in the study.

**Appendix E2 TACE treatment and response assessment**

Interventional radiologists with over 10 years of clinical experience have successfully performed conventional TACE (c-TACE) for all patients with HCC. The procedures for TACE strictly adhere to established treatment guidelines^[1]^. The standardized operational steps were as follows: (1) After administering local anesthesia in the right inguinal area, the modified Seldinger technique was used to puncture the right femoral artery. (2) A 5-F angiography catheter (RH catheter; Cook, Bloomington, IN) was selectively used for diagnostic angiography of the celiac trunk and superior mesenteric artery. (3) A 2.7-F microcatheter (Progreat; Terumo) was advanced to a super-selected tumor-supplying artery (hepatic or subhepatic), followed by the sequential infusion of oxaliplatin (75 mg/m²) and 5-fluorouracil (500 mg/m²) through the microcatheter. (4) Iodized oil (5–20 mL; Guerbet, Aulnay-sous-Bois, France) was mixed with epirubicin (30–50 mg/m²) and gelatin sponge, which served as an embolic agent, delivered under digital subtraction angiography (DSA; AlluraClarity FD 20, Philips).

The efficacy of the treatment was evaluated and documented using contrast-enhanced CT performed2 - 3 months after the first TACE treatment (the average time interval from the first treatment to the first efficacy evaluation in this study is 72 days) according to the Modified Response Evaluation Criteria in Solid Tumors (mRECIST). The treatment group members will decide whether to re-treat with TACE based on the evaluation results. After that, dynamic enhanced imaging examinations and liver function, alpha fetoprotein (AFP) tests will be performed every 6~12 weeks, and repeat TACE treatment will be performed "as needed" according to the efficacy evaluation (the median follow-up time within 6 months for all patients in this study was 2 times).

Patients treated with TACE were categorized into two groups based on their response: the TR group and the non-TR (nTR) group, as defined by the mRECIST^[2]^. The TR group included patients with a complete response (CR) or partial response (PR), while those with disease progression (PD) or stable disease (SD) were classified as nTR.

**Appendix E3 CT examinations and images preprocessing**

All of the patients enrolled in this group had similar scan settings, but with different systems and parameters. All patients were fasted overnight and drank 600-1000 ml of warm water about 20 minutes before the CT scan. CT scans are obtained when the entire liver area is covered and breath-holding in the axial plane. The iodized contrast media was injected into the antecubital vein with a pump injector at a rate of 3.5 mL/s, CT scans in the arterial phase and portal vein phase were obtained with a delay of 30 s and 60 s of contrast injection, respectively. The thickness and interval of the slice of the arterial phase and portal phase images are 5 mm. All images were reconstructed into images with 1.25 mm slice thickness and 1.25 mm spacing. Transfer all data to Picture Archiving and Communication Systems (PACS). The parameters for CT image acquisition are shown in **Table S1**.

Contrast-enhanced CT is the primary examination method for diagnosing and treating patients with HCC and is the most widely used imaging modality in Rad research. This study utilized two scanners for abdominal contrast-enhanced CT scans: the DISCOVERY CT750 (GE Healthcare, United States) and the SOMATOM Definition Flash CT (SOMATOM, Germany). All patients underwent multi-phase contrast-enhanced CT scans of the abdomen within two weeks prior to TACE treatment. The acquired data were transferred to an advanced workstation, where arterial phase CT images were retrieved from Picture Archiving and Communication Systems (PACS) for further evaluation. The CT images were exported from the PACS database in digital imaging and communication in medicine (DICOM) format. All DICOM images were then converted to the neuroimaging informatics technology initiative (NIFTI) format using Python software (version 3.7) and ITK-SNAP software (version 4.2.0; <http://www.itksnap.org>).

**Appendix E4 Feature extraction methodology**

The radiomics feature extraction in this study included three steps: Image standardization, deep learning feature and handcrafted feature extraction, respectively. Note that, all features were normalized by z-score method into a standardized value range.

**E4.1 Image standardization**

Image standardization was performed to transform the original image to a relatively standardized type for each CT phase to reduce the data variability among multi-center cohorts and to generate appropriate input for quantitative radiomics feature calculation. A two-step process for image standardization was implemented in this study: (a) all CT images were resampled to a voxel size of 1×1×1mm^3^ using cubic spline interpolation; and (b) the pixel intensity was normalized to transform the images to standardized inputs, which had the intensity range from -1024 to 1024 HU and the unified abdomen window (window-level [WL] of 50 HU and window-width [WW] of 350 HU).

**E4.2 Selection of radiomics features and construction of the radiomics signature**

All images are preprocessed, including image normalization, radiomics features and deep learning feature extraction. It is worth noting that all features are normalized to a standard range through the Z-score method. In order to improve accuracy and stability, according to the mRECIST evaluation criteria, the response after the first TACE treatment was independently evaluated by two experienced radiologists (A and B) and the arterial phase CT images were performed by these two doctors independently. Regions of interest (ROIs) were manually segmented to ensure intra- and inter-observer segmentation repeatability. The two doctors had 8 and 12 years of experience respectively and were blinded to the histopathological results. The segmented ROI did not include liver tissue surrounding the tumor. Each ROI area is fused to generate three-dimensional volumes of interest (VIOs). Finally, Professor C, who has more than 20 years of work experience, reviewed these sketches and revised the problematic sketches.

Radiomic features in VOI were extracted through the Pyradiomics (v.3.0.1) package. A total of four types of features were obtained: (1) first-order statistical features; (2) two-dimensional features based on shape; (3) texture features based on statistics: gray level co-occurrence matrix (GLCM) features, gray level run length matrix ( GLRLM) features, gray size zone matrix (GLSZM) features, adjacent gray tone difference matrix features (NGTDM) features, gray level dependency matrix (GLDM) features; (4) Use logarithm, gradient, square, square root, wavelet A total of 7 different categories of filters, exponential and local three-dimensional binary pattern (LBPD), generate corresponding derivative images on the original image. For each filtering and decomposition, first-order statistical features and statistics-based texture features are calculated. Finally, a total of 1834 quantitative radiomic features were extracted for each VOI.

The extracted radiomic features were first standardized using the Z-score method and then subjected to dimensionality reduction. To prevent model overfitting, feature screening is performed on the training set and Rad-signature is constructed. It includes the following steps: (1) Use Pearson correlation analysis. Features with a correlation greater than 0.9 are highly repetitive. Only one of them is retained and other redundant repeated features are discarded. (2) The filtered features are analyzed using univariate analysis (p< 0.05) Further screen out features; (3) Use the least absolute shrinkage and selection operator (LASSO) to perform feature screening to obtain highly relevant radiomic features; (4) Use the backward stepwise selection method in accordance with Akaike's information criterion (AIC), build a multivariable logistic regression model. And the predicted TR prediction probability value of each HCC patient was calculated and defined as Radiomics signature.

**Appendix E5 DL features extraction and selection**

Based on the arterial phase CT image, the window width was set to 350 Hounsfield units, and the window level was set to 50 HU. We selected the maximum 2D ROI plane of the tumor for analysis. Before training the Convolutional Neural Networks (CNN), we applied grayscale transformation to the CT images, standardized the background information, removed image noise, and enhanced the data. A small square with a pixel size of 224 × 224 was generated to decode tumor information from the CT images using a DL model. To address the substantial computational power required for training complex DL models, we utilized transfer learning technology with the ImageNet dataset to train a DenseNet-121 model. We removed the fully connected layer and softmax layer from the DenseNet-121 architecture, using the output of the final layer as DL features. Ultimately, the DenseNet-121 model produced 1,024 DL features from each CT image, which were then compressed into 32 features for the training set. These features were screened to construct a DL model, and the predicted probability of each HCC patient's TR was defined as the DL signature.

The extracted Rad features and DL features were fused, and the intra-class/inter-class correlation coefficients (ICC) were calculated to evaluate the repeatability of the features. An ICC greater than 0.9 indicated strong intra- and inter-observer agreement. We conduct univariate analysis using the appropriate independent t-test or Mann-Whitney U test based on the variable type, retaining features with a p-value of less than 0.05. The LASSO method was then used to further identify significant features. Finally, a deep learning Rad (DLR) model was constructed through multivariable LR analysis.

**Appendix E6**

In the comparison of Deep Learning + Radiology model (DLR), we filtered from logistic regression, SVM, MLP, and random forest models. Although the random forest model performs well in the training set, the prediction ability is poor in the test set, and the calibration curve is unstable. In comparison, the performance of the logistic regression model in these four models is relatively stable, so we choose the logistic regression model for the next step. The variables of the DLR model adopt pre-fusion, and the Signature value is calculated to be combined with the clinical. That is, the final Clinical+ Deep learning +Radiomics (CDLRN) comprehensive model is constructed to build Nomogram.

**References**

[1] GABA R C, LOKKEN R P, HICKEY R M, et al. Quality Improvement Guidelines for Transarterial Chemoembolization and Embolization of Hepatic Malignancy[J]. Journal of Vascular and Interventional Radiology, 2017, 28(9):1210-1223.e3.doi:10.1016/j.jvir.2017.04.025

[2] LLOVET J M, LENCIONI R. mRECIST for HCC: Performance and novel refinements[J]. Journal of Hepatology, 2020, 72(2):288-306.doi:10.1016/j.jhep.2019.09.026

**2. Supplementary Figures**


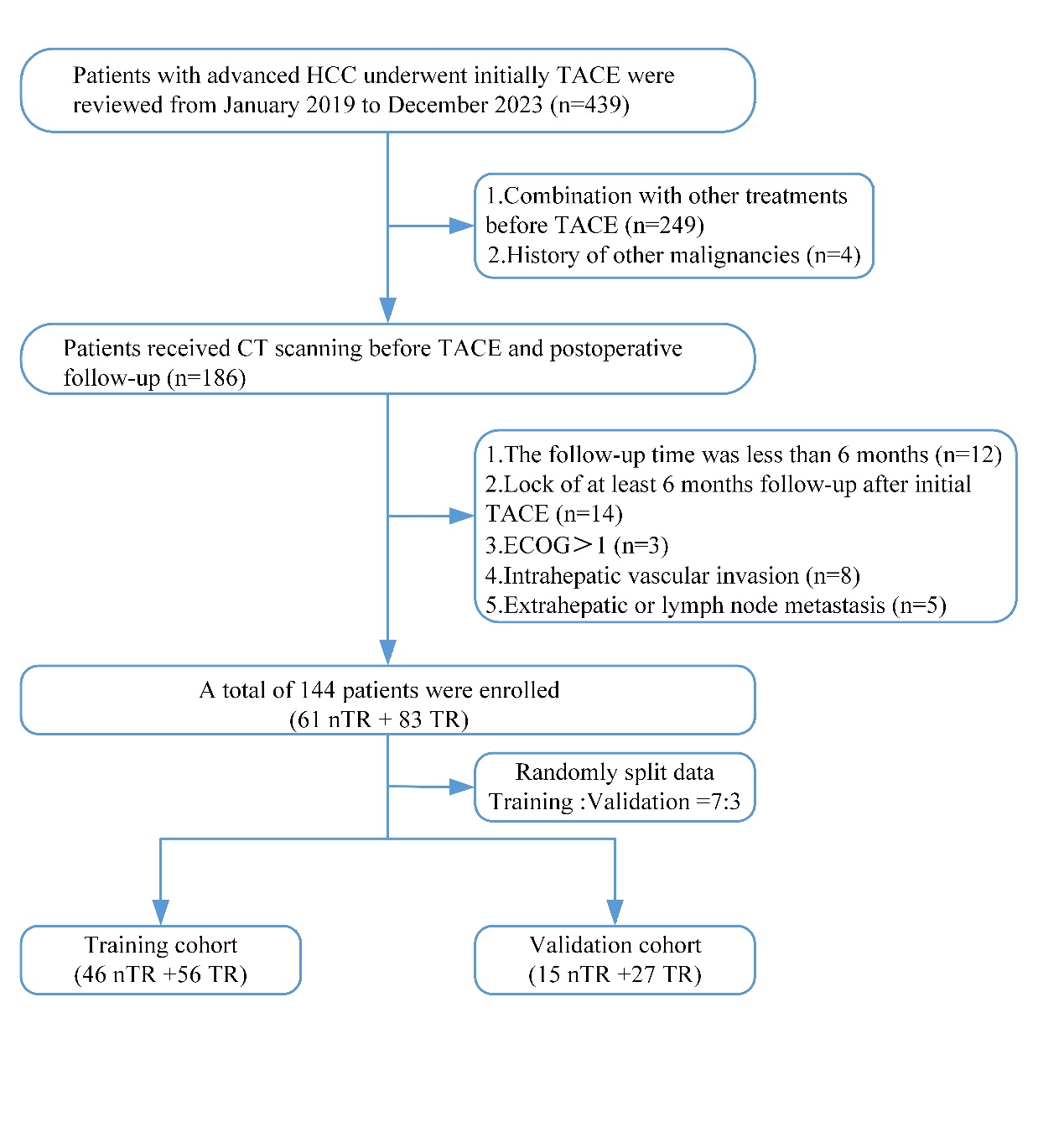


**Figure S1**. Flowchart of the patient selection process. HCC: Hepatocellular carcinoma; TACE: transcatheter arterial chemoembolization; TR: TACE response; nTR: non-TACE response


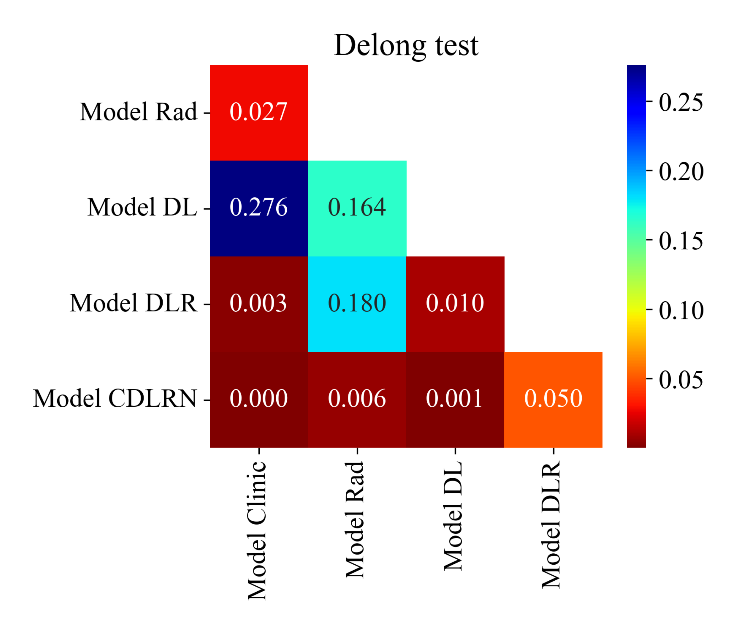


**Figure S2**. All models are tested by Delong

**3. Supplementary Tables**

**CT protocols**

All patients in the hospital underwent contrast-enhanced CT within 2 weeks before surgery. Table S1 shows the technical details of the CT protocols.

**Table S1. CT protocols**

| **Parameters** |  |  |
| --- | --- | --- |
| CT version | Discovery (CT750 HD  GE Healthcare, USA) | SOMATOM  Definition Flash (Siemens  Healthineers, Germany) |
| CT tube voltage | 120 kV | 120kv |
| CT tube current | Automatic tube-current | 290-330 mA |
| CT rotation time | 0.6 s | 0.5 s |
| CT detector collimation | 64×0.625 mm | 128×0.6 mm |
| section thickness | 5 mm | 5mm |
| section interval | 5 mm | 5mm |
| Image matrix | 512×512 | 512×512 |
| Contrast agent type | Omnipaque | Ultravist |
| Contrast agent concentration | 300mgI/mL | 350mgI/mL |
| Contrast agent dosage | infused 1.5ml/kg body weigh | infused 1.2ml/kg body weigh |
| Contrast agent infused rate | 3.5mL/s | 3.5mL/s |
| Arterial phase interval time | 30s after the contrast injection | 30s after the contrast injection |
| Reconstruction  image thickness for  Arterial phase | 1.25mm | 1.25mm |

| **Rad-score** | **Features** | **Coefficient** |
| --- | --- | --- |
| Radiomics | (Intercept) | 0.549 |
| signature |  |  |
|  | lbp_3D_k_firstorder_Kurtosis | -0.026 |
|  | lbp_3D_k_ngtdm_Complexity | -0.019 |
|  | lbp_3D_m2_firstorder_Range | -0.065 |
|  | lbp_3D_m2_glrlm_LongRunLowGrayLevelEmphasis | -0.032 |
|  | lbp_3D_m2_glszm_GrayLevelVariance | 0.073 |
|  | log_sigma_1_0_mm_3D_glszm_ZonePercentage | 0.001 |
|  | og_sigma_3_0_mm_3D_gldm_LargeDependenceLowGrayLevelEmphasis | 0.008 |
|  | logarithm_firstorder_Median | 0.024 |
|  | wavelet_HHL_firstorder_Skewness | -0.062 |
|  | wavelet_HHL_glcm_Correlation | 0.099 |
|  | wavelet_HLH_firstorder_Median | 0.083 |
|  | wavelet_HLL_glszm_ZoneVariance0.055 | -0.015 |
|  | wavelet_LLH_firstorder_90Percentile | 0.055 |
|  | wavelet_LLL_glcm_Imc2 -0.007541 | 0.016 |
|  | wavelet_LLL_glszm_ZoneVariance | -0.008 |
| DL signature | (Intercept) | 0.549 |
|  | DL_2 | -0.01 |
|  | DL_13 | -0.001 |
|  | DL_24 | -0.025 |
|  | DL_30 | -0.081 |

**Table S2. Results of radiomics feature selection and Rad-score building**

**Abbreviations:** XL, original image filtered directionally with low-pass filter along x and y directions; XH, original image filtered directionally with high-pass filter along x and y directions; DLx, xth feature map; H, histogram feature; GLRLM, GLRLM feature.
